# Real-time myoelectric control of wrist/hand motion in Duchenne muscular dystrophy: A case study

**DOI:** 10.3389/frobt.2023.1100411

**Published:** 2023-04-06

**Authors:** Kostas Nizamis, Anıl Ayvaz, Noortje H. M. Rijken, Bart F. J. M. Koopman, Massimo Sartori

**Affiliations:** ^1^ Systems Engineering and Multidisciplinary Design Group, Department of Design, Production, and Management, Faculty of Engineering Technology, University of Twente, Enschede, Netherlands; ^2^ Neuromechanical Modelling and Engineering lab, Department of Biomechanical Engineering, Faculty of Engineering Technology, University of Twente, Enschede, Netherlands; ^3^ Research Group Smart Health, Saxion University of Applied Sciences, Enschede, Netherlands

**Keywords:** admittance, Duchenne muscular dystrophy, forearm, intention decoding, myoelectric control, pattern recognition, surface electromyography, wrist

## Abstract

**Introduction:** Duchenne muscular dystrophy (DMD) is a genetic disorder that induces progressive muscular degeneration. Currently, the increase in DMD individuals' life expectancy is not being matched by an increase in quality of life. The functioning of the hand and wrist is central for performing daily activities and for providing a higher degree of independence. Active exoskeletons can assist this functioning but require the accurate decoding of the users' motor intention. These methods have, however, never been systematically analyzed in the context of DMD.

**Methods:** This case study evaluated direct control (DC) and pattern recognition (PR), combined with an admittance model. This enabled customization of myoelectric controllers to one DMD individual and to a control population of ten healthy participants during a target-reaching task in 1- and 2- degrees of freedom (DOF). We quantified real-time myocontrol performance using target reaching times and compared the differences between the healthy individuals and the DMD individual.

**Results and Discussion:** Our findings suggest that despite the muscle tissue degeneration, the myocontrol performance of the DMD individual was comparable to that of the healthy individuals in both DOFs and with both control approaches. It was also evident that PR control performed better for the 2-DOF tasks for both DMD and healthy participants, while DC performed better for the 1-DOF tasks. The insights gained from this study can lead to further developments for the intuitive multi-DOF myoelectric control of active hand exoskeletons for individuals with DMD.

## 1 Introduction

Duchenne muscular dystrophy (DMD) is the most common form of muscular dystrophy in male children, affecting 1 in 4,000 individuals worldwide (Opstal et al., 2014). DMD is caused by a gene mutation that compromises the production of dystrophin protein, the absence of which causes progressive weakness in the skeletal, respiratory and cardiac muscles. This leads to severe physical disability and shortened life expectancy (Nowak and Davies, 2004). Boys with DMD become increasingly dependent on external aids in their daily activities due to the progressive paresis and the loss of functional ability (Haley et al., 2010). However, over the last two decades, life expectancy has improved significantly due to improvements in healthcare, with the current estimate being around 40 years (Bushby et al., 2010). This has led to a significant increase in the number of DMD adults living with severe physical impairments who have a strong dependency on care (Rahbek et al., 2005).

Functional interaction with the world heavily relies on hand manipulation, a central element for every individual in performing the activities of daily living (ADL) (Lue et al., 2016). However, the dynamic daily support of hand functioning in individuals with DMD remains a challenge (Wagner et al., 2007).

Here, wearable robotic devices, such as hand exoskeletons, can provide a solution (8). A recent study showed that the overnight use of passive hand orthoses helps preserve the passive range of motion in terms of wrist extension and thumb abduction (Weichbrodt et al., 2018). The usage of active hand exoskeletons could further assist DMD individuals in terms of tackling a greater range of motor tasks (Bartels et al., 2011) as this would enable dynamic movements with the active participation of the user (Lobo-Prat et al., 2017a).

For the intuitive and robust control of active hand exoskeletons, accurate decoding of the user’s intention is the primary challenge (Lenzi et al., 2012). A clinically viable way to enable robust control involves the use of surface electromyography (sEMG) (Farina and Sartori, 2016; Geethanjali, 2016; Negro et al., 2016; Negro and Orizio, 2017). Various sEMG-based control methods have been developed to decode the hand motor intention of the user, with direct control (DC) (Rahman et al., 2001; Lenzi et al., 2012) and pattern recognition (PR)-based control (Parker et al., 2006) being the most common. While regression (Muceli et al., 2012) and model-based approaches (Sartori et al., 2016; Durandau et al., 2018) are being developed, they are not yet broadly considered to be clinical standards. DC is broadly used with upper extremity prostheses (Parker and Scott, 1986; Williams, 1990), while common PR classification methods include linear discriminant analysis (LDA), support vector machines (SVM), fuzzy approaches, regression and multi-layer perceptron (MLP) (Dellacasa Bellingegni et al., 2017).

Importantly, there is a limited amount of systematic analyses on the feasibility of forearm sEMG as a source of control signals for active hand exoskeletons in individuals with DMD (Nizamis et al., 2020). However, two studies involving participants suffering from other forms of muscular dystrophy (Vogel et al., 2013; Polygerinos et al., 2015) showed promising results in terms of the functional decoding of motor intention from the hand/wrist. Meanwhile, the performance of sEMG control was recently compared to force control with an active planar support for the shoulder and elbow in DMD individuals (Lobo-Prat et al., 2017a). Here, it was shown that both methods are able to decode intended arm movements. However, the possibility of decoding wrist-hand movements was not explored. Our previous research, showed promising results when trying to characterize offline the neuromotor profile of the forearm of DMD individuals using PCA analysis in combination with high-density EMG (Nizamis et al., 2020), as well as for the real time control of a robotic hand exoskeleton using DC control over 1 degree of freedom (DOF) (Bos et al., 2019).

In this paper, we make the first attempt to evaluate the real-time sEMG decoding of wrist-hand motor intention of one DMD individual for PR and DC control for 1-, and 2- DOF movements. We compare sequential DC and PR as potential sEMG control methods and provide an analysis of their differences, during a target-reaching task in 1- and 2-DOF. For this study, our PR method incorporates an MLP, while both our approaches combine myocontrol with a first-order admittance model (Lobo-Prat et al., 2017a), which allows for the manipulation of the interface virtual dynamics and a subsequent further tailoring of the control across all the participants. This is beneficial, especially in terms of the participant with DMD, who is expected to have different assistance requirements than the healthy participants. Real-time myocontrol, admittance modelling and out-of-the-lab use are central requirements for the function-related use of assistive technology in DMD sufferers’ everyday lives.

## 2 Materials and methods

### 2.1 Participants

The experiment was carried out with ten healthy adults (seven males and three females) aged between 20 and 33 who have no hand-related impairment, and one male adult with DMD of age 25 who is unable to use his hands in terms of simple tasks such as, for example, holding a pen (Brooke score of 6 out of 6 (Jung et al., 2012)). The DMD individual consistently experiences early onset fatigue and extensive hand/wrist related contractures. The Medical Ethics Committee of Twente approved the study design, the experimental protocol and the procedures, while all the participants were fully informed about the study through a letter and subsequently provided written informed consent (Protocol number: NL59061.044.16). Each participant took part in one session, where they performed the proposed target-reaching task with both myocontrol methods. The healthy participants serve as a baseline of the relative feasibility of the proposed methods for the individual with DMD.

### 2.2 Experimental setup and signal acquisition

**FIGURE 1:**
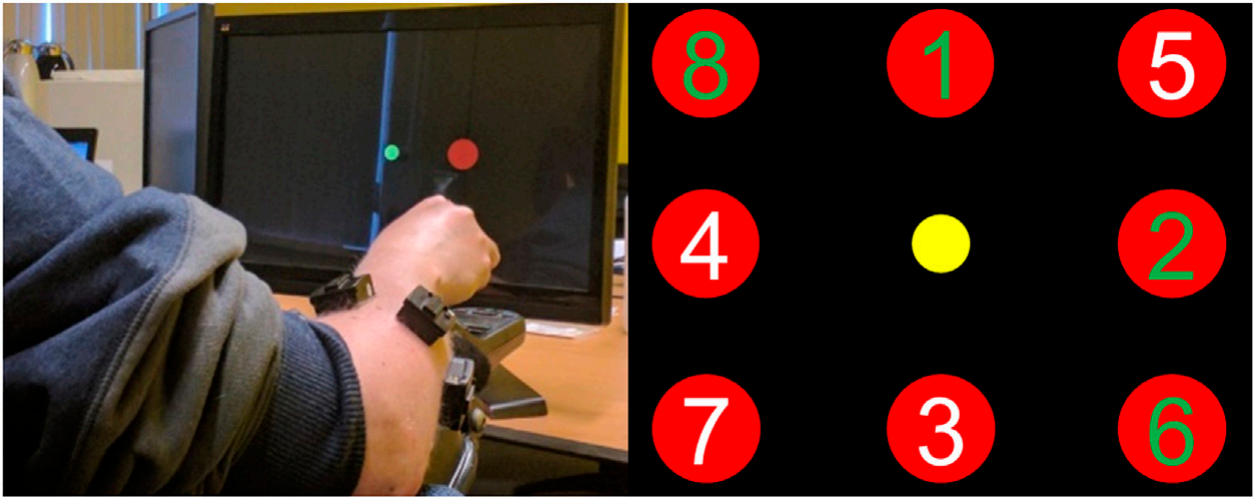
On the left side, the participant with Duchenne muscular dystrophy controlling a virtual cursor in 1 and 2 degrees-of-freedom, while resting his arm on his wheelchair. The wireless EMG bipolar electrodes can be seen around his forearm. On the right side, the locations of all possible targets (red) shown by their target number, and the cursor (yellow). Targets 1-4 are part of the 1-DOF trials and targets 5-8 of the 2-DOF. Each trial accepted as successful when participant kept the cursor inside the target for 2 s. The trials with a green number (1,2,6, and 8) are those performed by the individual with DMD.

The experimental setup is shown in Figure 1, left. During the experiment, each healthy participant was seated in a chair in front of a computer screen, with their forearm placed on a soft foam-padded arm support on the table, in a neutral position. Meanwhile, the DMD participant was similarly positioned, while his arm rested on the arm support of his wheelchair, in a supinated position due to contractures not allowing the neutral position (Figure 1, left). Six dry, active bipolar sEMG electrodes (Trigno Lab, Delsys, United States) were placed around the dominant forearm of each of the participants.

**FIGURE 2:**
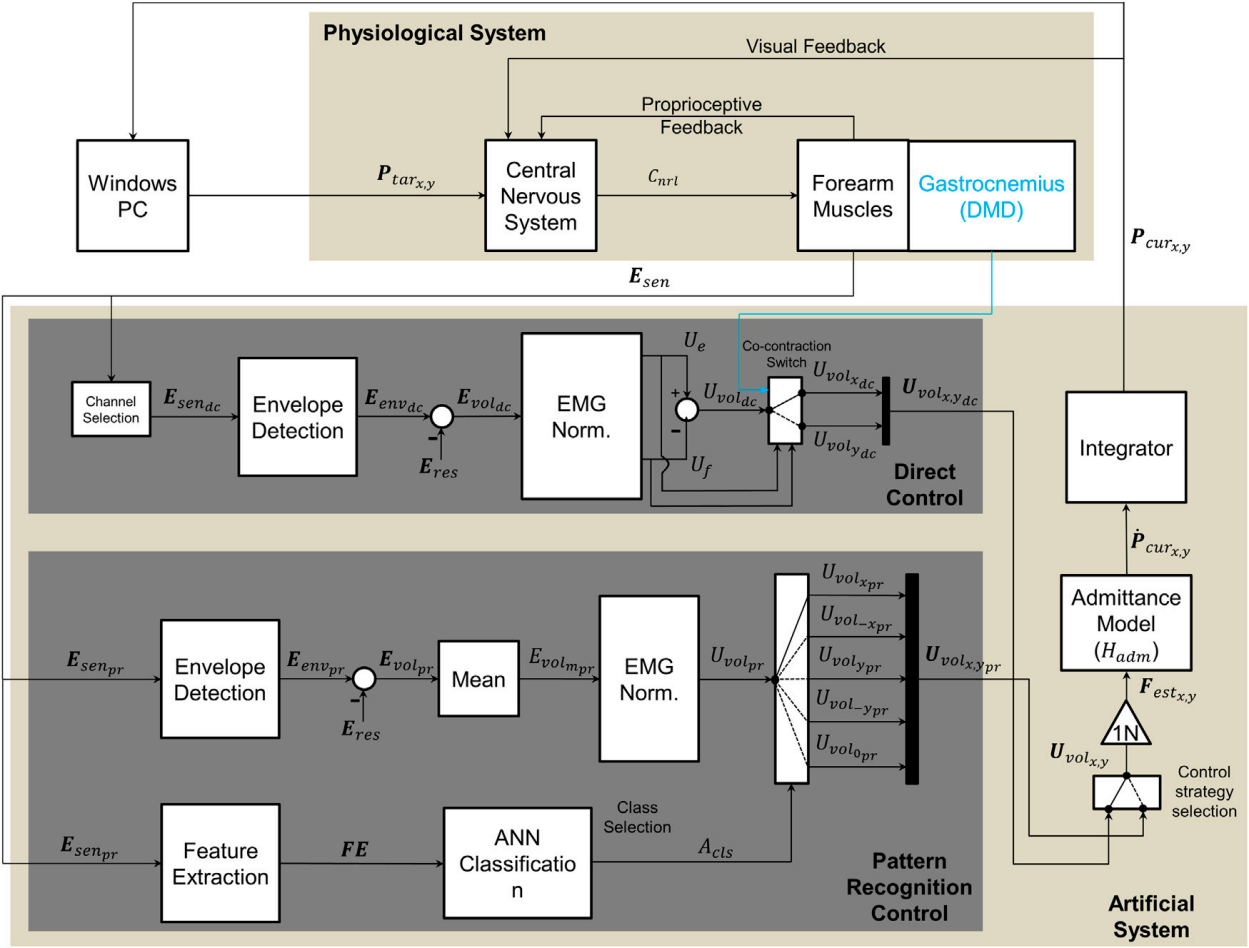
Diagram of implemented control methods, adapted from Lobo-Prat et al. (Lobo-Prat et al., 2017a). Bold font style symbols indicate vectors and regular font style symbols indicate scalars. The upper section represents the physiological system (participant), while the lower section represents the experimental system. To perform a movement participant first see the target on the screen (**P**_tarx,y_). The target is generated by a python script running in the host computer. This generates a neural command (C_nrl_) with their central nervous system, which results in muscle activation at forearm muscles where sEMG signals (**E**_sen_) are measured. Intention of the user is decoded from these sEMG signals. In direct control the sEMG signals (**E**_sendc_) are measured from agonist/antagonist muscle pair from forearm (Flexor Carpi Ulnaris/Extensor Carpi Ulnaris) and the resting sEMG (**E**_res_) is subtracted to acquire the voluntary sEMG (**E**_voldc_). The signal is normalized to the maximum voluntary contraction (MVC) and control signals are generated from each muscle (U_e_,U_f_). A voluntary control signal (U_voldc_) is obtained by subtracting the control signal of the flexor muscle from that of the extensor muscle (reverse for left handed participants). A co-contraction switch, was used to alternate DOF. In case of the DMD participant, an electrode in the gastrocnemius was used to switch (blue line). In pattern recognition control sEMG signals (**E**_senpr_) are measured from six electrodes placed on forearm (hexagonal grid). Time domain features (FE) were extracted from measured sEMG signals and these features were then used by ANN classifier to identify the movement class (Acls). This class is then used to select the final control signal (**U**_volx,ypr_). This control signal (U_volpr_) is the normalized mean envelope of the six electrodes. In both control methods the estimated voluntary forces (F_estx,y_) are used as input to a first order admittance model (H_adm_) that resembles the dynamics of a mass-damper system. The resulting velocity of the cursor ( $\dot{P}$ _curx,y_) is send to an integrator (**P**_curx,y_) and then to the windows PC to control the position of the cursor on the screen. This motion was sensed by the participants proprioception and by visual feedback and was used to generate new neural commands to reach new target positions (**P**_tarx,y_).

Firstly, one electrode was placed on the muscle belly of the flexor carpi ulnaris (FCU), and one on the muscle belly of the extensor carpi ulnaris (ECU). Due to the difficulties of people with DMD to independently activate muscle groups (found in our previous work (Nizamis et al., 2020) and also *via* pilot tests with the participant of this study), we decided to implement a mode switch between DOFs when it comes to DC control, inspired by Wurth et al. (Wurth and Hargrove, 2014). The co-contraction of the FCU and the ECU was used in order to switch between DOF during the 2-DOF DC for the healthy participants. Meanwhile, the other four electrodes were placed in between, equidistantly, while for the DMD participant, an extra electrode was added to his gastrocnemius muscle. This was used as a trigger to switch between DOFs during 2-DOF DC (blue line in Figure 2), since he could not co-contract his forearm muscles in a controlled fashion and without experiencing severe fatigue. Prior to the electrode placement, the skin was cleaned with alcohol to ensure optimal electrode-skin impedance, while the sEMG signals were obtained through the use of a real-time computer (xPC Target 5.1, MathWorks Inc., United States). The analogue-to-digital conversion was performed using a National Instruments card (PCI-6229, National Instruments Corp., United States) at a sampling frequency of 1 kHz and with a 16-bit resolution. A National Instruments USB-data-acquisition device (6259, National Instruments Corp., United States) was used to record the offline data to train the MLP. The controllers were running on a real-time computer and were sending position commands through UDP/IP communication to a Windows PC in order to control the position of the cursor in the screen.

### 2.3 Experimental protocol

A screen-based target-reaching task was employed in this study to evaluate the performance of the two myocontrol methods in 1-, and 2-DOF. The experiment consisted of one session with two parts, one for each of the different myocontrol methods compared. Both DC and PR were coupled with an admittance model (see section 2.4). At the beginning of each part, the maximum voluntary contraction (MVC) of each participant was recorded during isometric contractions. MVC was acquired as the mean envelope signal over a period of 3 s of contraction. Afterwards, participants were instructed to relax their muscles, and the researcher acquired the average of the processed sEMG envelope signal during the last 3 s of rest. This was used later to calculate voluntary sEMG (see Eq. 1). For each type of controller, tasks were performed both in1-DOF and 2-DOF (Figure 1, right). Both the 1-DOF tasks and the 2-DOF tasks included four target locations. For targets 1-4, the participants had to move only in 1-DOF for every trial, while for targets 5-8 they had to sequentially move in 2-DOF (Figure 1, right). Between tasks, the participants were provided with rest periods of five to 10 min, depending on the reported experienced muscle fatigue. Each task consisted of eight targets with ten trials per target. Meanwhile, each target appeared ten times and the order of appearance is shown in Figure 1, right.

The order of the evaluation of the myocontrol methods was randomised across the participants in order to avoid order effects in the results. Each trial began with the appearance of a target on the screen, where the participants were then instructed to move the cursor as fast as possible from its initial to the target position and keep it there for 2 s. The cursor returned to the initial position upon trial completion and the next trial would then start in 2 s. The participants first familiarised themselves with each myocontrol method before starting each part. For every target, the first two trials were discarded and were not included in the analysis to account for learning.

For the DMD individual, the experiment was conducted including only targets 1, 2, 6, 8 (2 for 1-DOF and 2 for 2-DOF, Figure 1, right). This was dictated by the need to comply with ethically viable standards in terms of avoiding the onset of extensive contractures that would result in pain. The reduced subset of targets was chosen in order to capture the maximum variability of movements (1-DOF targets 1 and 2 required opposite movements, as did 2-DOF targets 6 and 8).

### 2.4 Myoelectric control

Before performing a movement, the participants were presented with a target appearing on the screen. Then they generated a neural command within their nervous system, which resulted in the subsequent activation of their forearm muscles (**E**_sen_) that was measured *via* dry surface bipolar sEMG electrodes. Raw sEMG signals were digitally filtered with a second-order Butterworth high-pass filter with a 20 Hz cut-off frequency to reduce any movement artefacts.

The envelopes were calculated through full-wave rectification of the signal and the subsequent application of a fourth-order Butterworth low-pass filter with a 2 Hz cut-off frequency (Lenzi et al., 2012). The envelopes were normalised to the MVC. Normalised-filtered sEMG signals were then used to create the control signal (U_volx,y_) for both the pattern recognition (PR) method and the direct control (DC) method, as shown in Figure 2.

#### 2.4.1 DC method

Both DOF on the *x*-axis and the *y*-axis were controlled using the sEMG from an antagonistic muscle pair (see Table 1). Here, two out of the six electrodes that were placed on the forearm were used. The one on the muscle belly of the FCU, and the one on the muscle belly of the ECU. The envelopes of the sEMG signals (**E**_envdc_) were computed and the average of the processed sEMG envelope signal during rest (**E**_rest)_ was subtracted from them to acquire the voluntary sEMG signals (**E**_voldc_) and divided by the MVC to acquire the voluntary control signals ((**U**_e,f_)) for flexion or extension, that were finally subtracted from each other to acquire the final direction of the control signal (Uvoldc), according to the following equations:

$$E_{voldce,f}=E_{envdce,f}-E_{reste,f}$$ (1)

**TABLE 1:** Mapping of limb motion to cursor motion during PR and DC myocontrol.

MyocontrolMethod	Participant	Cursor left	Cursor right	Cursor up	Cursor down
**Pattern** **Recognition**	Right Handed (S1-S5, S7-S10)	Wrist Flexion	Wrist Extension	Hand Open	Hand Closed
	Left Handed (S6)	Wrist Extension	Wrist Flexion	Hand Open	Hand Closed
	DMD	Hand Closed	Hand Open	Wrist Extension	Wrist Flexion
**Direct** **Control**	Right Handed (S1-S5, S7-S10)	Wrist Flexion	Wrist Extension	Wrist Flexion	Wrist Extension
	Left Handed (S6)	Wrist Extension	Wrist Flexion	Wrist Extension	Wrist Flexion
	DMD	Wrist Extension	Wrist Flexion	Wrist Extension	Wrist Flexion

and

$$U_{e,f}=\frac{E_{voldce,f}}{E_{mvcdce,f}}$$ (2)

$$U_{voldc}=U_{e}-U_{f}$$ (3)

Finally, mode switching between different DOFs was achieved through the co-contraction of the FCU and the ECU (the threshold of co-contraction was adjusted to a comfortable level for each subject during the familiarization time). For the participant with Duchenne muscular dystrophy (DMD), an extra electrode was added to his gastrocnemius muscle. This was used as a trigger to switch between DOFs (blue line Figure 2), since he could not co-contract his forearm muscles in a controlled fashion and without experiencing fatigue. The use of the switch determined the final voluntary normalised control signal (**U**_volx,ydc_) that served as input to the admittance model.

#### 2.4.2 PR method

A pattern recognition artificial neural network (ANN) myocontrol method was implemented using MATLAB’s Neural Network Toolbox (The MathWorks Inc., Natick, MA) for the following motion classes: hand open and close; wrist flexion and extension; and no motion. Each motion class corresponded to a different DOF movement of the cursor (see Table 1). The method chosen in this study was a multilayer perceptron method–which is one of the most popular PR classification methods (Ortiz-Catalan et al., 2013) since it yields high classification accuracy compared to other commonly used PR methods (Dellacasa Bellingegni et al., 2017) – with one hidden layer consisting of ten neurons.

For the training of the supervised classification algorithm, sEMG signals were collected prior to the PR session during five repetitions of 2-s comfortable contractions for each motion class. The classifier was trained with the use of five commonly used time-domain features: root mean square, mean absolute value, number of zero crossings, slope sign changes and waveform length (Hudgins et al., 1993). The features were extracted using a window of 250 ms (which is within an acceptable range for real-time myoelectric applications) (Smith et al., 2011) with an overlap of 125 ms. Similar to the DC methods signal processing the envelopes of all six electrodes were computed (E_envpr_) and the resting sEMG (E_res_) was subtracted to calculate the voluntary sEMG (E_volpr_). The voluntary sEMG envelopes of all six electrodes were first averaged (E_volmpr_) (Eq. 4), and then normalised to the mean of the envelopes of the six electrodes during the MVC (Eq. 5) to create a control signal proportional to the overall muscle activity:

$$E_{volmpr}=\frac{{\sum_{1}^{6}E}_{voldc}}{6}$$ (4)

$$U_{volpr}=\frac{E_{volmpr}}{E_{mvcmpr}}$$ (5)

The input to the admittance model (**U**_volx,ypr_) was a vector with five elements (motion classes). One element was equal to the U_volpr_ and the remainder equal to zero (depending on which motion class was decoded by the classifier (A_cls_).

For both myocontrol methods, the participants practiced the target-reaching task prior to the experiment in order to grasp the motion mapping (Table 1). In the case of the ANN, the machine learning algorithm was re-trained in case any participant was not comfortable with the control of the cursor (low responsiveness, misclassifications and fatigue). This occurred 1-2 times on average per participant (no difference between the healthy participants and the one with DMD was noted).

### 2.5 Admittance model

Both myocontrol methods were used in combination with a first-order admittance model (*H*_*adm*_) (Eq. (6)), which received the sEMG estimated control signal **U**_volx,y_ multiplied by a conversion gain of 1N (Figure 2) in order to acquire the estimated force (F_estx,y_), which served as input to a first-order admittance model. The output was the cursor velocity (**Ṗ**_curx,y_).

$$H_{adm}=\frac{{\dot{P}}_{curx,y}\left(s\right)}{F_{estx,y}\left(s\right)}=\frac{1}{As+B}$$ (6)

where A is the virtual inertia related parameter, B is the virtual damping related parameter, and s is the Laplace transform variable. For the healthy participants, the parameters of the admittance model were fixed *a priori* based on pilot trials and were left unchanged for all of them (Section 3.2). Meanwhile, for the DMD participant, the parameters were fine tuned. While we initially asked the DMD participant to perform the experiment with the same parameters as the healthy participants, this proved to be too fatiguing for him. Subsequently, we adjusted the parameters according to his feedback through trial and error, in order to achieve an acceptable compromise between less fatiguing control and precision of the cursor.

### 2.6 Data analysis

**FIGURE 3:**
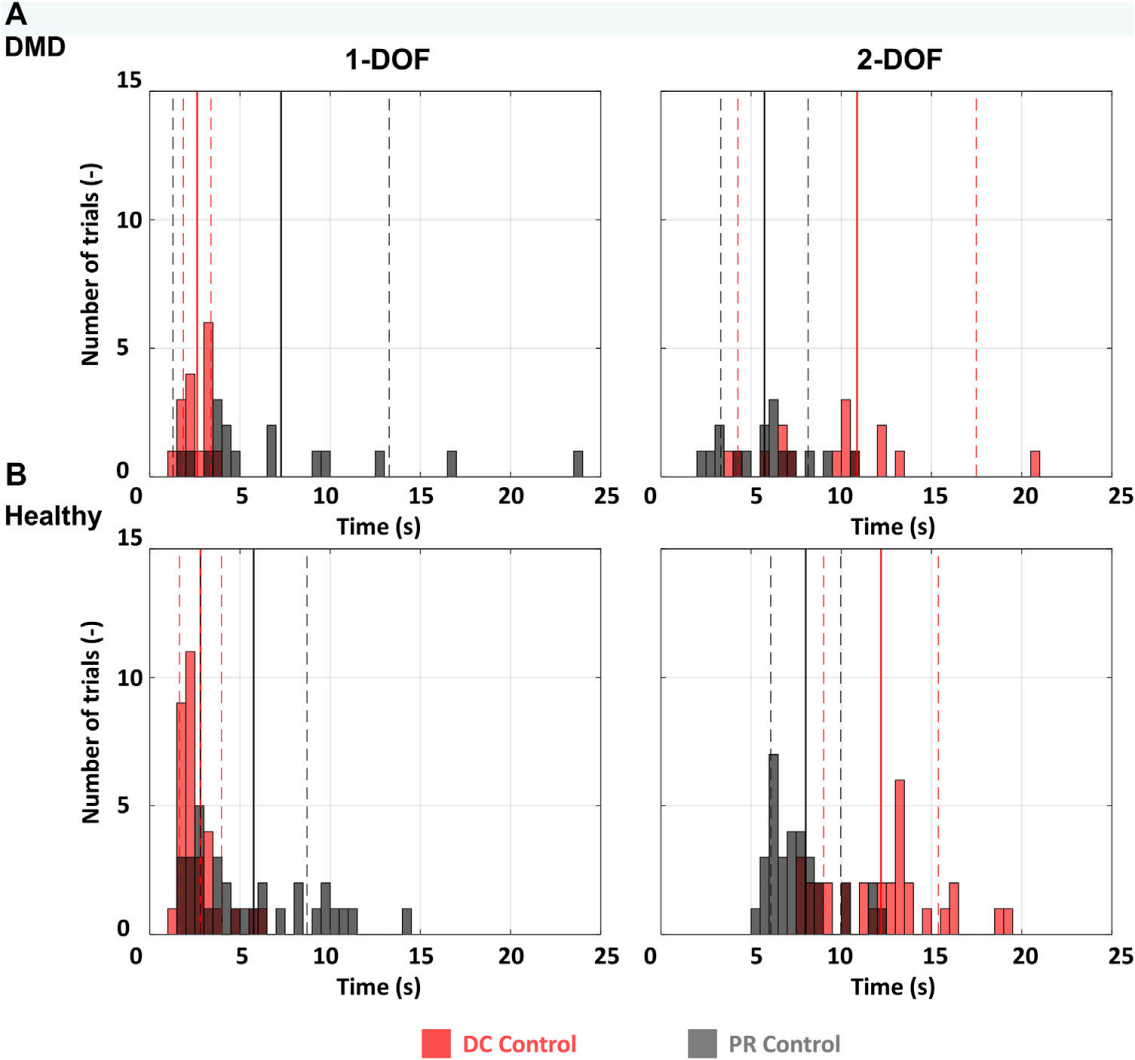
Histograms for all participants. 1-DOF and 2-DOF tasks were compared separately. **(A)** The distribution of reaching times for all 1-DOF and 2-DOF tasks for the participant with DMD. Since every DOF includes two targets and each target was performed 8 times, we have 16 events per histogram. **(B)** The average distribution of reaching times for all 1-DOF and 2-DOF tasks for the ten healthy participants. Since every DOF includes four targets and each target was performed 8 times, we have 32 events per histogram. The full vertical lines represent the mean and the dashed the standard deviation.

Reaching time was used to analyze the reaching performance of the participants (The dataset, including all reaching times for all participants, is available online (Nizamis et al., 2022). Reaching time was defined as the time needed to reach the target as it appeared on the screen, starting from the moment the target appeared. The 2 s of settling time, inside the target were not included in the reaching time. The performance metrics were averaged across all healthy participants for every trial of every target per session. Since the statistical comparison between one participant with DMD and ten healthy would not be valid, we performed descriptive statistics (mean and standard deviation), and compared the data using histograms of the distribution of reaching times in all trials (Figure 3) for healthy and DMD. participants. The healthy data are the results of the average of all 10 participants for all trials (N = 32, for each DOF and each control method, as they performed four targets eight times per DOF). For the participant with DMD N = 16 (as he performed half the trials compared to the healthy controls).

## 3 Results

### 3.1 Healthy vs. DMD

This subsection presents the results of the comparison between the reaching times of the DMD participant and those of the healthy population for both myocontrol methods and types of tasks.

As illustrated in Figure 3, the participant with DMD exhibited comparable reaching times to the average of the healthy controls for both the 1-DOF and the 2-DOF tasks. For the 1-DOF tasks the participant with DMD achieved an average reaching time of 2.6 ± 0.8 s (mean ± SD) for DC control and 7.3 ± 6 s for PR, while the average time of the healthy participant for the same tasks was 2.8 ± 1.2 s for DC control, and 5.8 ± 3 s for PR (Figure 3). For the 2-DOF tasks the same can be observed in Figure 3. The participant with DMD, achieved on average a reaching time of 10.9 ± 6.6 s for DC control and 5.7 ± 2.4 s for PR. For the same tasks, the average time of the healthy participants was 12.2 ± 3.2 s for DC control, and 8 ± 1.9 s for PR.

When it comes to the different control methods, the reaching time during DC control for the participant with DMD is lower than that with PR for 1-DOF tasks, while the opposite is observed for the 2-DOF tasks. The exact same pattern is found also when looking at the healthy participants reaching time data, with their DC control performance being better for 1-DOF tasks and worse for 2-DOF tasks.

### 3.2 Admittance model personalization

In our approach we differentiated the parameters for the admittance model for the healthy controls and the DMD participant. The latter was more comfortable with a lower virtual inertia related parameter than the healthy population (A = 6.6⋅10^−4^ and A = 5⋅10^−4^ respectively). The virtual damping-related parameter was higher for the participant with DMD (B = 6⋅10^−4^) than for the healthy population (B = 4⋅10^−4^).

## 4 Discussion

In this case study, inspired by our previous work on the offline feasibility of myocontrol for people with DMD (Nizamis et al., 2020), we tested for the first time two very commonly used myocontrol methods combined with an admittance model and evaluated them among ten healthy participants and one participant with DMD using virtual target-reaching tasks. Despite the muscular degeneration, the DMD individual displayed a comparable myocontrol performance in relation to the healthy individuals. Moreover, our proposed admittance model enabled the setting of appropriate virtual dynamics for the DMD participant, facilitating a myocontrol capacity catered to the patient’s needs. This suggests that a personalised myocontrol scheme can successfully decode intention in DMD sufferers despite the degeneration in the underlying muscle tissues.

The participant with DMD was able to control the cursor on the screen with success using both the DC and the PR methods. However, for mode switching in DC, the participant with DMD used a switch placed on the gastrocnemius muscle, since controlling the switch *via* the co-contraction of his forearm muscles was rather fatiguing. Same as the healthy participants, the DMD individual exhibited lower reaching times while using DC for 1-DOF and PR for 2-DOF. This result has been observed previously with amputees in similar tasks (Jiang et al., 2014; Wurth and Hargrove, 2014; Young et al., 2014). While the reaching times of the DMD participant for 1- and 2-DOF were similar to those of the healthy participants, this might be attributed to the fact that the former performed a reduced version of the experimental protocol used for the healthy population, which allowed for comparable cognitive and physical demands. Additionally, the different switching mode may have given an advantage to the participant with DMD, as mode switching can be difficult and confusing for novel myocontrol users (Jiang et al., 2014). Nevertheless, the results suggest that the DMD participant was able to perform the requested tasks and that both myocontrol methods were both comfortably and successfully used. This is a promising result for the further investigation of the presented myocontrol methods as potential ways to decode hand/wrist motor intention in people with DMD, since the successful decoding of their intention will enable them to control active hand exoskeletons. Additionally, since this subject has the highest Brooke score (6 out of 6) we assume that despite our sample size of 1, our results about the feasibility of myocontrol can be generalised to more people with DMD.

The use of an admittance model in combination with sEMG can provide an advantage for DMD, since it offers an additional level of customisation that is absent in most conventional myoelectric control methods. Table 1 shows that the individual with DMD required a different level of assistance than the healthy participants. In fact, the former preferred a lower parameter A (related to the virtual mass) and a higher parameter B (related to the virtual damping). This enabled him to move the cursor in a less fatiguing way (lower virtual mass) and to achieve stable myocontrol (higher virtual damping). As we found in our previous study (Nizamis et al., 2020), the absolute amplitude of the sEMG of people with DMD is considerably lower than this of healthy participants and they present a lower amount of independent muscle activations in the forearm. Therefore, we expect that in future studies, a person-specific adaptation of both parameters (virtual mass and damping-related parameters) will be required in order to allow some adjustment in terms of the level of the individual needs of each subject with DMD, which can vary according to the level of disease progression and the rehabilitation measures received.

In line to what was found in previous similar studies with amputees (Jiang et al., 2014; Wurth and Hargrove, 2014), the switching between the DOFs that was required for DC control appeared to be unintuitive for a number of the healthy participants, which was reflected in the higher reaching times for DC during the 2-DOF tasks. Hence, the reaching times for DC may have been slightly underestimated, given that PR allowed the participants to perform uninterrupted movements. The appearance order of the targets during the target-reaching tasks for both DOFs and the myocontrol methods was not randomised (they always appeared sequentially in their numerical order: 1-4 for 1-DOF and 5-8 for 2-DOF). While this may have created a learning effect throughout the experiment, we do not believe this presents a major concern since the directions of the targets were alternating one after another, and, as a result, the participants had to perform different movements to reach the target. Moreover, this setting was applied in all the conditions tested, meaning it affected them all in equal measure.

The participant with DMD consistently experienced early fatigue onset throughout the tests. However, the modification in the protocol ensured that enough trials were performed with the appropriate variability for extracting useful insights while ensuring any ethical requirements were met. Non-etheless, our research was limited due to the low number of available participants with DMD, which made it difficult to recruit multiple participants. Hence, our conclusions must be regarded with some caution as a higher number of participants would be required to ensure they are appropriately robust.

Future work should involve an evaluation of the effect of forearm orientation in hand/wrist motor intention decoding with individuals with DMD. Despite its limitations, our study indicates that for the decoding of simple 1-DOF motions of the hand, DC demonstrates better performance than PR. This feasibility of DC control for the real-time myocontrol for a 1-DOF hand exoskeletons was also demonstrated in our previous work (Bos et al., 2019). As it has also become clear by previous research, sEMG may be the most feasible way of controlling assistive devices as the disease progresses (Lobo Prat, 2016). Despite the loss in muscle strength, sEMG is retained in DMD even in late stages of the disease (Lobo-Prat et al., 2017b). In contrast, in terms of 2-DOF tasks, DC performs worse than PR. In future work we will implement these myocontrol methods and validate their use with an active hand exoskeleton for multiple DOFs (Bos et al., 2018; Bos et al., 2019).

## Data Availability

The datasets presented in this study can be found in online repositories. The names of the repository/repositories and accession number(s) can be found below: https://data.mendeley.com/datasets/tn8zn77fh5.

## References

[B1] Bartels B. Pangalila R. F. Bergen M. P. Cobben N. A. M. Stam H. J. Roebroeck M. E. (2011). Upper limb function in adults with Duchenne muscular dystrophy. J. Rehabil. Med. 43 (9), 770–775. 10.2340/16501977-0841 21826385

[B2] Bos R. A. Nizamis K. Koopman B. F. J. M. J. M. Herder J. L. Sartori M. Plettenburg D. H. (2019). A case study with SymbiHand: An sEMG-controlled electrohydraulic hand orthosis for individuals with duchenne muscular dystrophy. IEEE Trans. Neural Syst. Rehabilitation Eng. 28 (1), 258–266. 10.1109/tnsre.2019.2952470 31825868

[B3] Bos R. A. Nizamis K. Plettenburg D. H. Herder J. L. (2018). “Design of an electrohydraulic hand orthosis for people with duchenne muscular dystrophy using commercially available components,” in 2018 7th IEEE International Conference on Biomedical Robotics and Biomechatronics (Biorob), Enschede, Netherlands, 26-29 August 2018 (IEEE), 305–311.

[B4] Bushby K. Finkel R. Birnkrant D. J. Case L. E. Clemens P. R. Cripe L. (2010). Diagnosis and management of duchenne muscular dystrophy, part 1: Diagnosis, and pharmacological and psychosocial management. Lancet Neurology 9, 77–93. 10.1016/s1474-4422(09)70271-6 19945913

[B5] Dellacasa Bellingegni A. Gruppioni E. Colazzo G. Davalli A. Sacchetti R. Guglielmelli E. (2017). NLR, MLP, SVM, and LDA: NLR, MLP, SVM, and LDA: A comparative analysis on EMG data from people with trans-radial amputation. J. Neuroeng Rehabil. 14 (1), 82. 10.1186/s12984-017-0290-6 28807038 PMC5557564

[B6] Durandau G. Farina D. Sartori M. (2018). Robust real-time musculoskeletal modeling driven by electromyograms. IEEE Trans. Biomed. Eng. 65 (3), 556–564. 10.1109/tbme.2017.2704085 28504931

[B7] Farina D. Sartori M. (2016). “Surface electromyography for man-machine interfacing in rehabilitation technologies,” in Surface electromyography: physiology, engineering and applications 2nd ed. Editor Farina D. Merletti R. (IEEE/Wiley), 540–560.

[B8] Geethanjali P. (2016). Myoelectric control of prosthetic hands: State-of-the-art review. Med. Devices Evid. Res. 9, 247–255. 10.2147/mder.s91102 PMC4968852 27555799

[B9] Haley S. M. Coster W. I. Kao Y. C. Dumas H. M. Fragala-Pinkham M. A. Kramer J. M. (2010). Lessons from use of the pediatric evaluation of disability inventory: Where do we go from here? Pediatr. Phys. Ther. 22, 69–75. 10.1097/pep.0b013e3181cbfbf6 20142708 PMC3631526

[B10] Hudgins B. Parker P. Scott R. N. (1993). A new strategy for multifunction myoelectric control. IEEE Trans. Biomed. Eng. 40 (1), 82–94. 10.1109/10.204774 8468080

[B11] Jiang N. Rehbaum H. Member S. Vujaklija I. Graimann B. (2014). Intuitive, online, simultaneous, and proportional myoelectric control over two degrees-of-freedom in upper limb amputees, IEEE Trans. 22 (3), 501–510. 10.1109/tnsre.2013.2278411 23996582

[B12] Jung I. Y. Chae J. H. Park S. K. Kim J. H. Kim J. Y. Kim S. J. (2012). The correlation analysis of functional factors and age with Duchenne muscular dystrophy. Ann. Rehabil. Med. 36, 22. 10.5535/arm.2012.36.1.22 22506232 PMC3309314

[B13] Lenzi T. de Rossi S. M. M. Vitiello N. Carrozza M. C. (2012). Intention-based EMG control for powered exoskeletons. IEEE Trans. Biomed. Eng. 59 (8), 2180–2190. 10.1109/tbme.2012.2198821 22588573

[B14] Lobo Prat J. (2016). Control interfaces to actively support the arm function of men with Duchenne Muscular Dystrophy. [PhD Thesis - Research UT, graduation UT, University of Twente]. Universiteit Twente. 10.3990/1.9789036541701

[B15] Lobo-Prat J. Janssen M. M. H. P. Koopman B. F. J. M. Stienen A. H. A. de Groot I. J. M. (2017). Surface EMG signals in very late-stage of duchenne muscular dystrophy: A case study. J. Neuroeng Rehabil. 14 (1), 86. 10.1186/s12984-017-0292-4 28851391 PMC5576133

[B16] Lobo-Prat J. Nizamis K. Janssen M. M. H. P. Keemink A. Q. L. Veltink P. H. Koopman B. F. J. M. (2017). Comparison between sEMG and force as control interfaces to support planar arm movements in adults with duchenne: A feasibility study. J. Neuroeng Rehabil. 14 (1), 73. 10.1186/s12984-017-0282-6 28701169 PMC5508565

[B17] Lue Y. J. Chen S. S. Lu Y. M. (2016). Quality of life of patients with duchenne muscular dystrophy: From adolescence to young men. Disabil. Rehabil. 0 (0), 1–6. 10.1080/09638288.2016.1196398 27347814

[B18] Muceli S. Farina D. Member S. S. S. Farina D. Member S. S. S. Farina D. (2012). Simultaneous and proportional estimation of hand kinematics from EMG during mirrored movements at multiple degrees-of-freedom. IEEE Trans. Neural Syst. Rehabilitation Eng. 20 (3), 371–378. 10.1109/tnsre.2011.2178039 22180516

[B19] Negro F. Orizio C. (2017). Robust estimation of average twitch contraction forces of populations of motor units in humans. J. Electromyogr. Kinesiol. 37, 132–140. 10.1016/j.jelekin.2017.10.005 29101911

[B20] Negro F. Yavuz U. Ş. Farina D. (2016). The human motor neuron pools receive a dominant slow-varying common synaptic input. J. Physiology 594 (19), 5491–5505. 10.1113/jp271748 PMC5043036 27151459

[B21] Nizamis K. Koopman B. Rijken N. H. M. Ayvaz A. Sartori M. (2022). Data for: Real-time myoelectric control of wrist/hand motion in Duchenne muscular dystrophy: A case study. Mendeley Data, V1. 10.17632/tn8zn77fh5.1 PMC10116050 37090893

[B22] Nizamis K. Rijken N. H. M. van Middelaar R. Neto J. Koopman B. F. J. M. Sartori M. (2020). Characterization of forearm muscle activation in duchenne muscular dystrophy via high-density electromyography: A case study on the implications for myoelectric control. Front. Neurol. 11, 231. 10.3389/fneur.2020.00231 32351441 PMC7174775

[B23] Nowak K. J. Davies K. E. (2004). Duchenne muscular dystrophy and dystrophin: Pathogenesis and opportunities for treatment: Third in molecular medicine review series. EMBO Rep. 5, 872–876. 10.1038/sj.embor.7400221 15470384 PMC1299132

[B24] Opstal S. L. S. H. V. Jansen M. van Alfen N. de Groot I. J. M. (2014). Health-related quality of life and its relation to disease severity in boys with duchenne muscular dystrophy: Satisfied boys, worrying parents - a case-control study. J. Child. Neurol. 29 (11), 1486–1495. 10.1177/0883073813506490 24141275

[B25] Ortiz-Catalan M. Brånemark R. Håkansson B. (2013). BioPatRec: A modular research platform for the control of artificial limbs based on pattern recognition algorithms. Source Code Biol. Med. 8, 11. 10.1186/1751-0473-8-11 23597283 PMC3669028

[B26] Parker P. A. Scott R. N. (1986). Myoelectric control of prostheses. Crit. Rev. Biomed. Eng. 13 (4), 283–310. 3512166

[B27] Parker P. Englehart K. Hudgins B. (2006). Myoelectric signal processing for control of powered limb prostheses. J. Electromyogr. Kinesiol. 16 (6), 541–548. 10.1016/j.jelekin.2006.08.006 17045489

[B28] Polygerinos P. Galloway K. C. Sanan S. Herman M. Walsh C. J. (2015). “EMG controlled soft robotic glove for assistance during activities of daily living,” in IEEE International Conference on Rehabilitation Robotics, Singapore, 11-14 August 2015 (IEEE), 55–60.

[B29] Rahbek J. Werge B. Madsen A. Marquardt J. Steffensen B. F. Jeppesen J. (2005). Adult life with Duchenne muscular dystrophy: Observations among an emerging and unforeseen patient population. Pediatr. Rehabil. 8, 17–28. 10.1080/13638490400010191 15799132

[B30] Rahman T. Ramanathan R. Stroud S. Sample W. Seliktar R. Harwin W. (2001). Towards the control of a powered orthosis for people with muscular dystrophy. Proc. Institution Mech. Eng. Part H J. Eng. Med. 215, 267–274. 10.1243/0954411011535858 11436269

[B31] Sartori M. Llyod D. G. Farina D. (2016). Neural data-driven musculoskeletal modeling for personalized neurorehabilitation technologies. IEEE Trans. Biomed. Eng. 63 (5), 879–893. 10.1109/tbme.2016.2538296 27046865

[B32] Smith L. H. Hargrove L. J. Lock B. A. Kuiken T. A. (2011). Determining the optimal window length for pattern recognition-based myoelectric control: Balancing the competing effects of classification error and controller delay. IEEE Trans. Neural Syst. Rehabilitation Eng. 19, 186–192. 10.1109/tnsre.2010.2100828 PMC4241762 21193383

[B33] Vogel J. Bayer J. van der Smagt P. (2013). “Continuous robot control using surface electromyography of atrophic muscles,” in 2013 IEEE/RSJ International Conference on Intelligent Robots and Systems, Tokyo, Japan, 03-07 November 2013 (IEEE), 845–850.

[B34] Wagner K. R. Lechtzin N. Judge D. P. (2007). Current treatment of adult Duchenne muscular dystrophy. Biochim. Biophys. Acta 1772 (2), 229–237. 10.1016/j.bbadis.2006.06.009 16887341

[B35] Weichbrodt J. Eriksson B. M. Kroksmark A. K. (2018). Evaluation of hand orthoses in Duchenne muscular dystrophy. Disabil. Rehabil. 40 (23), 2824–2832. 10.1080/09638288.2017.1347721 28687062

[B36] Williams T. W. (1990). Practical methods for controlling powered upper-extremity prostheses. Assist. Technol. 2 (1), 3–18. 10.1080/10400435.1990.10132142 10149040

[B37] Wurth S. M. Hargrove L. J. (2014). A real-time comparison between direct control, sequential pattern recognition control and simultaneous pattern recognition control using a Fitts’ law style assessment procedure. J. Neuroeng Rehabil. 11 (1), 91. 10.1186/1743-0003-11-91 24886664 PMC4050102

[B38] Young A. J. Smith L. H. Rouse E. J. Hargrove L. J. (2014). A comparison of the real-time controllability of pattern recognition to conventional myoelectric control for discrete and simultaneous movements. J. NeuroEngineering Rehabilitation 11, 5. 10.1186/1743-0003-11-5 PMC3895741 24410948

